# Supplementary material for: Reproducibility in the UK biobank of genome-wide significant signals discovered in earlier genome-wide association studies
Source: Sci Rep. 2021 Sep 20;11:18625. doi: 10.1038/s41598-021-97896-y (PMC8452698; doi:10.1038/s41598-021-97896-y)
Supplement: Supplementary file 1 — Supplementary Information. [file 41598_2021_97896_MOESM1_ESM.pdf]

Appendix: Reproducibility in the UK Biobank of Genome-Wide Significant Signals Discovered in Earlier Genome-wide Association Studies

Table of contents

Extended methods **2**

Supplementary Figures **4**

eFigure 1 4

eFigure 2 5

eFigure 3 5

eFigure 4 6

eFigure 6 8

eFigure 7 9

Supplementary Tables **10**

eTable1 10

eTable2 10

eTable3 11

eTable4 12

PRISM Checklist 12

References **15**

Extended methods

We searched the GWAS Atlas (available at https://atlas.ctglab.nl/) for two, independent GWAS on the same trait conducted in Europeans (or results available exclusively for Europeans). The GWAS atlas is a publically available, and free collection of GWAS; it contains both GWAS conducted without UK Biobank (UKBB) data and also GWAS on all the traits available within the UBB. The authors of the GWAS atlas conducted 600 independent GWAS on all available traits from UK Biobank release 2 data (under application ID 16406) (1). The GWAS atlas contains 4,727 GWAS across 28 different domains, and allows users to download full summary statistics for all GWAS.

We searched the entirety of the GWAS atlas (by domain) for two GWAS on the same trait: an earlier GWAS using data not included in the UKBB (we termed this GWAS the 'discovery GWAS') and then a second, independent GWAS using exclusively UKBB data (we termed this GWAS the 'replication GWAS'). Further inclusion criteria was GWAS conducted in European subjects (or results available for exclusively Europeans) and GWAS with more than 50 genome-wide significant SNVs. We searched the GWAS atlas up to December 2019. We chose the GWAS atlas as this atlas contained GWAS containing only UKBB data (conducted by the authors), as well as other GWAS containing no UKBB data (not conducted by the authors).

Once two independent GWAS were found for the same trait, we identified SNVs present in both GWAS (via rsID) and only included SNVs shared between the two GWAS. We then identified SNVs reaching Genome-wide significance ($P<5e-8$) in the discovery GWAS and assessed if these SNVs were reproduced

in the replication GWAS (determined by P<5e-8 and effect size in a congruent direction e.g. OR both above 1). Before we assessed congruence of SNV effect direction we matched the effect alleles in both GWAS, if the effect allele differed between discovery and replication GWAS, we flipped the replication GWAS effect allele to match the discovery GWAS SNV effect allele. If we flipped the replication GWAS effect allele, we consequently inverted the SNV effect direction; i.e. for quantitative traits we made the SNV effect (β) negative if it was originally positive (or vice versa), and for binary traits we inverted the odds ratio: for example, an OR of 1.3 became 0.89 (1/1.3).

To determine the change in SNV effect size between the discovery GWAS and replication GWAS we perform three broad steps. First, to facilitate comparison of SNV effect sizes between discovery GWAS and replication GWAS for quantitative traits we standardised SNV effect sizes. The summary statistics publically available for UKBB GWAS (conducted by (1) were not standardised. Thus, with guidance with Watanabe (email correspondence January 2020), we standardized quantitative effect sizes (β) from Z-statistics as a function of MAF and sample size, via the formula from (2): $Standardised\,\beta = z/sqrt(2p(1-p)(n+z^2))$, where z is Z score converted from P-value, p is the minor allele frequency (MAF) and $n$is the sample size of the GWAS. Then, to further aid interpretation we aligned the SNV effect scale to an effect size per 1 standard deviation (SD). Standard deviations (SD) were taken from the UKBB for our quantitative traits (BMI, SBP, DBP, waist circumference, hip circumference, and pulse rate). Second we converted all SNV effect sizes to OR. For GWAS on binary traits (performed by logistic regression), these SNV effect sizes were already reported as OR (or, occasionally as log(OR), from which we calculated the OR (exp(OR)). However, for quantitative traits SNV effect sizes are reported as regression coefficients (β) from linear regression models. Chinn describes a method to convert linear regression coefficients to OR (3). This method was described to facilitate the meta-analysis of quantitative and binary effect sizes (similar to analyses performed in our

study). Chinn shows that linear regression coefficients can be converted to a log(OR) by multiplying by 1.81, from which the OR can be calculated by taking the exponential, e.g. $OR = exp((1.81\beta))$, where β is the linear regression coefficient (SNV effect size for quantitative trait, from linear regression). Third, to aid interpretation of our model, we converted all SNV effect sizes to positive. As all SNV effect sizes were now ORs, and ORs are symmetrical mathematical functions, we inverted OR that were below 1, via $1/OR$ (we did this for the same SNV in both the discovery and replication GWAS). We then constructed single predictor linear models as described in the main text. Lastly, we calculated the replication rate for each phenotype using a Bonferroni corrected P-value for the replication cohort P-value (instead of 5e-8), calculated by: P= 0.05/N, where N is the number of significant SNVs in discovery GWAS.

Supplementary Figures

eFigure 1

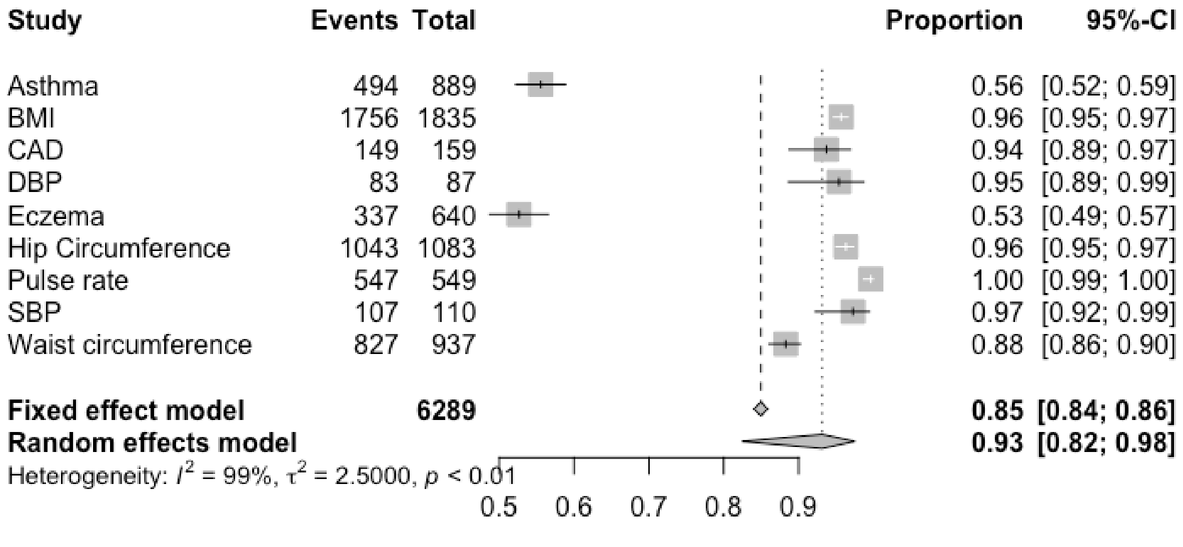

<u>eFigure 1</u>: Forest plot for SNV replication for all phenotypes

eFigure 2

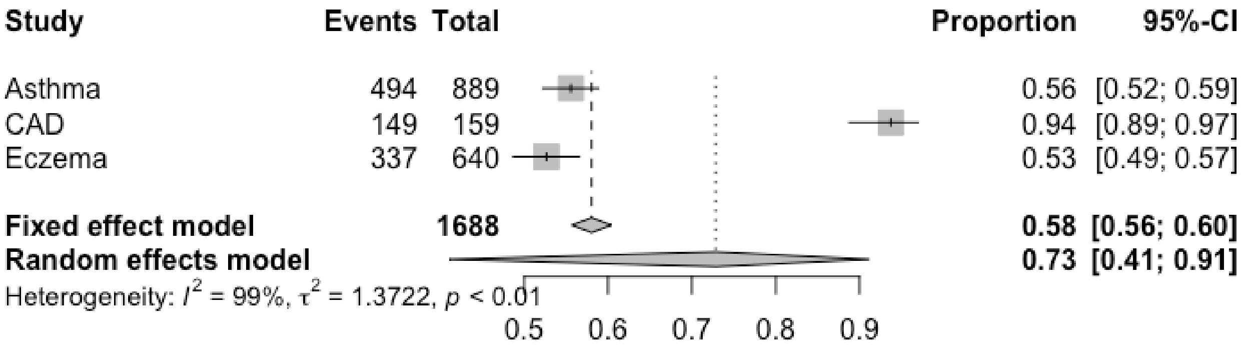

eFigure 2: Forest plot for SNV replication for binary phenotypes

eFigure 3

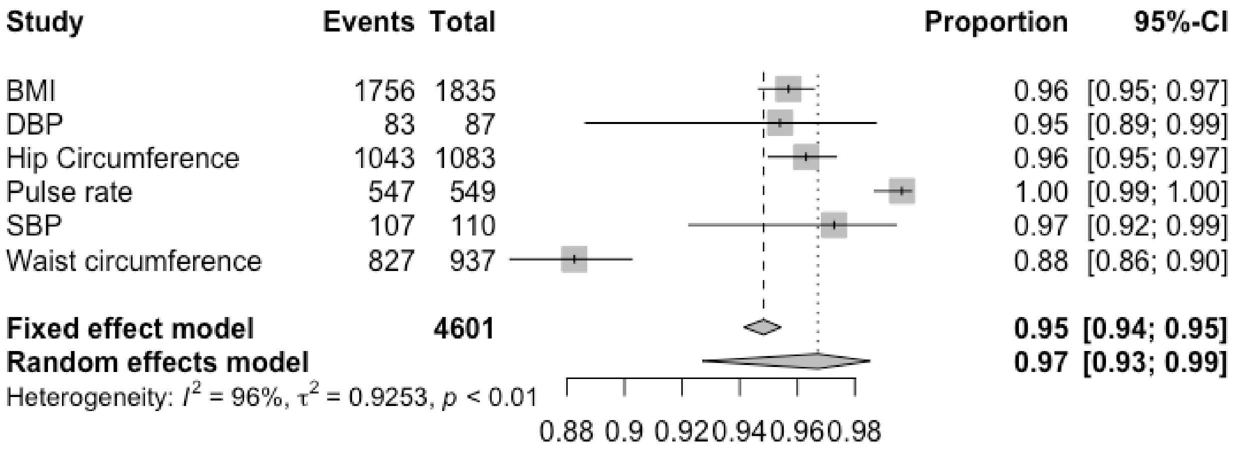

eFigure 3: Forest plot for SNV replication for quantitative phenotypes

eFigure 4

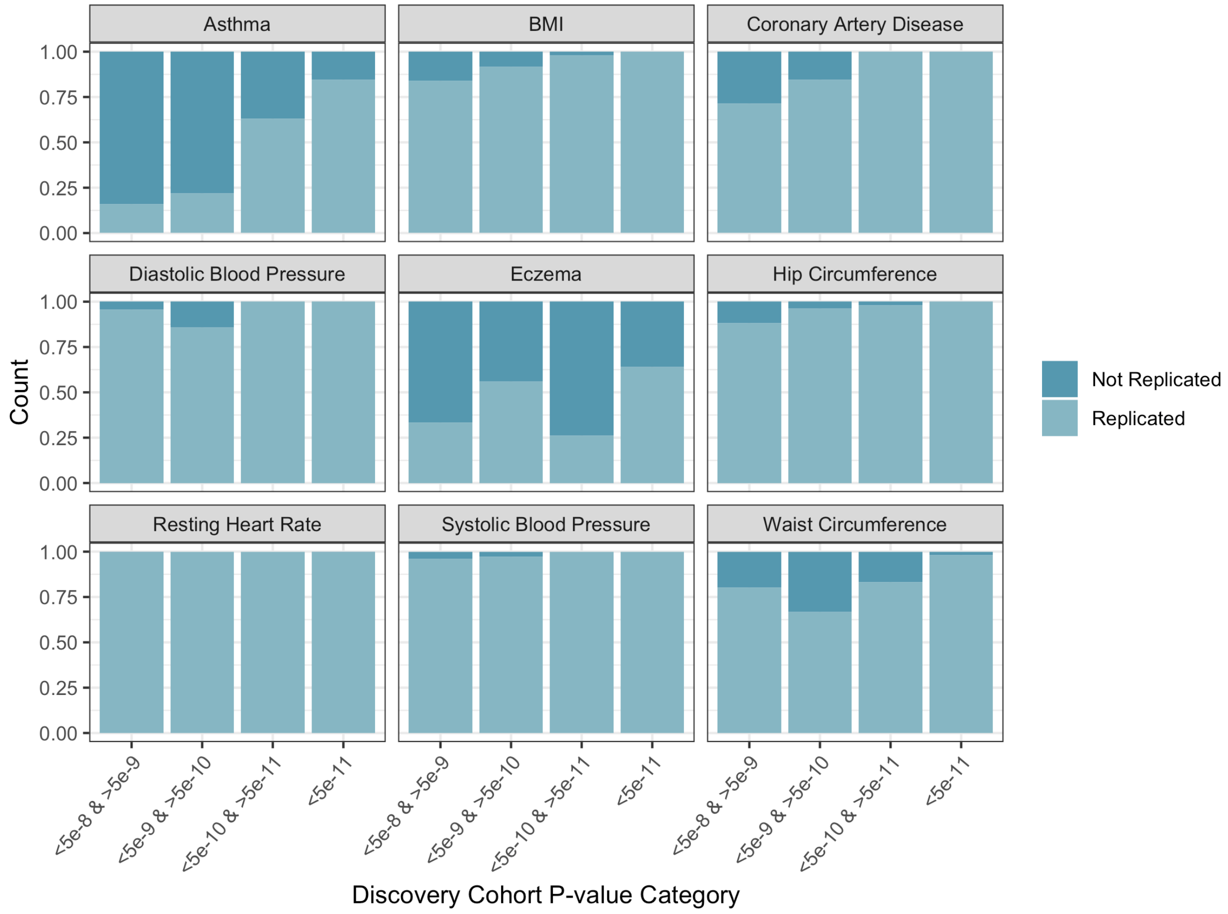

eFigure 4: SNV replication across phenotypes per P-value

eFigure 5

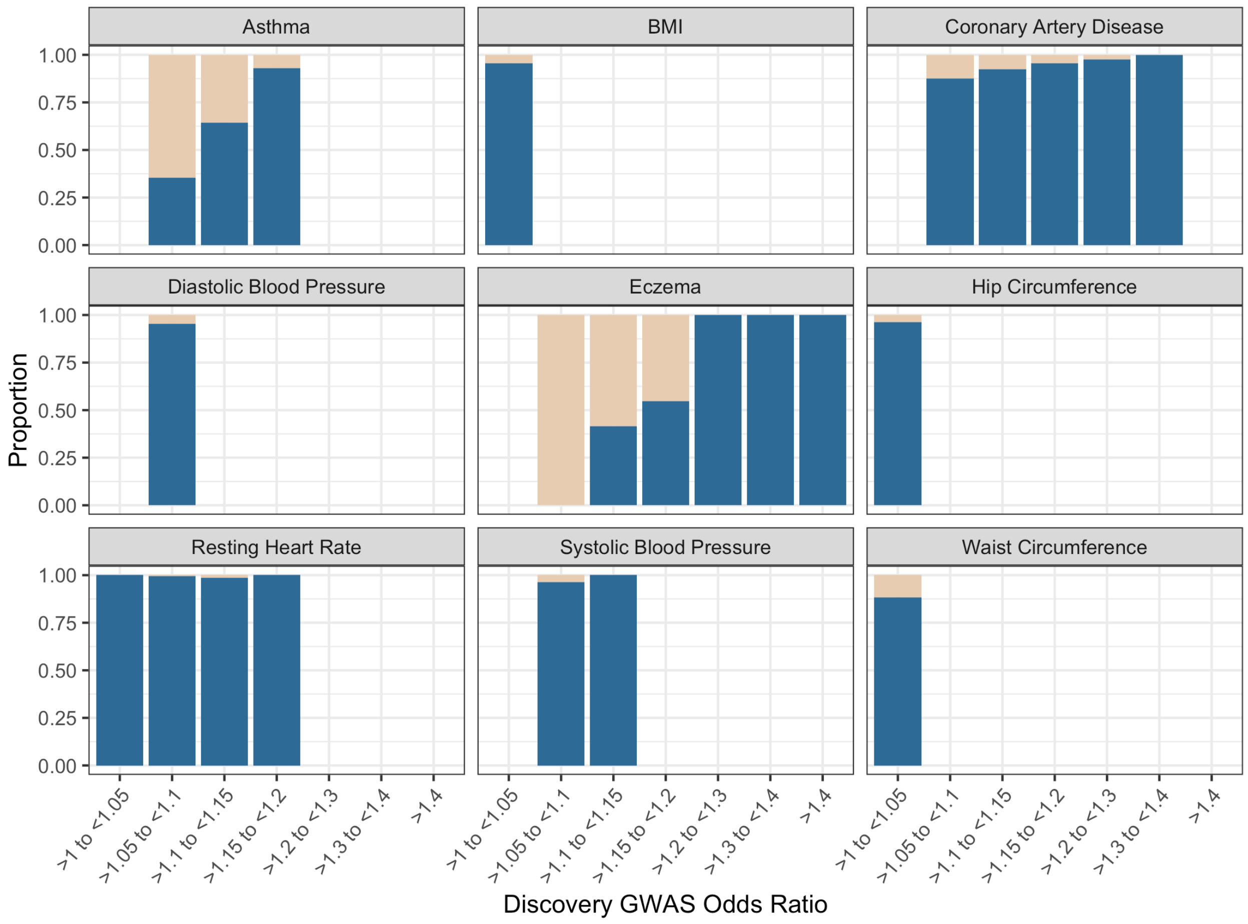

eFigure 5: SNV replication across phenotypes per Odds Ratio

eFigure 6

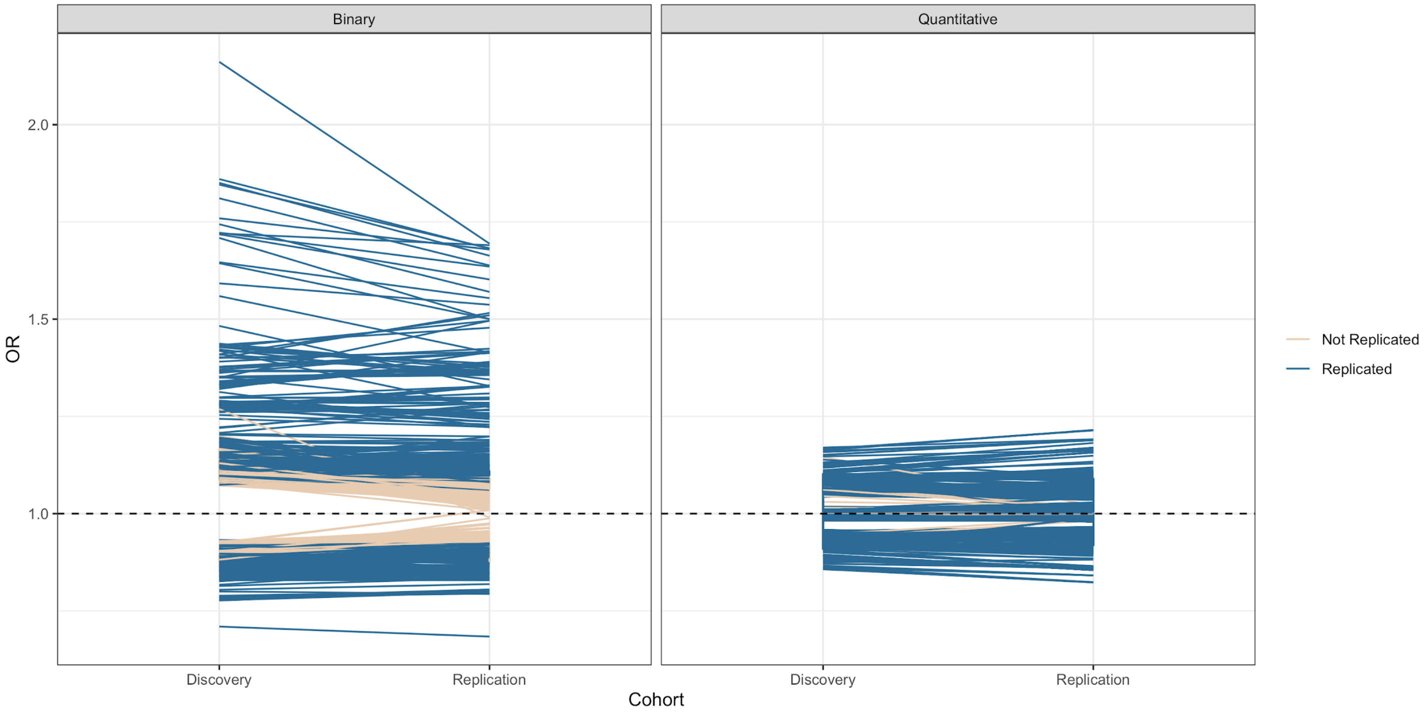

<u>eFigure 6:</u> SNV replication for binary and quantitative traits per Odds Ratio

eFigure 7

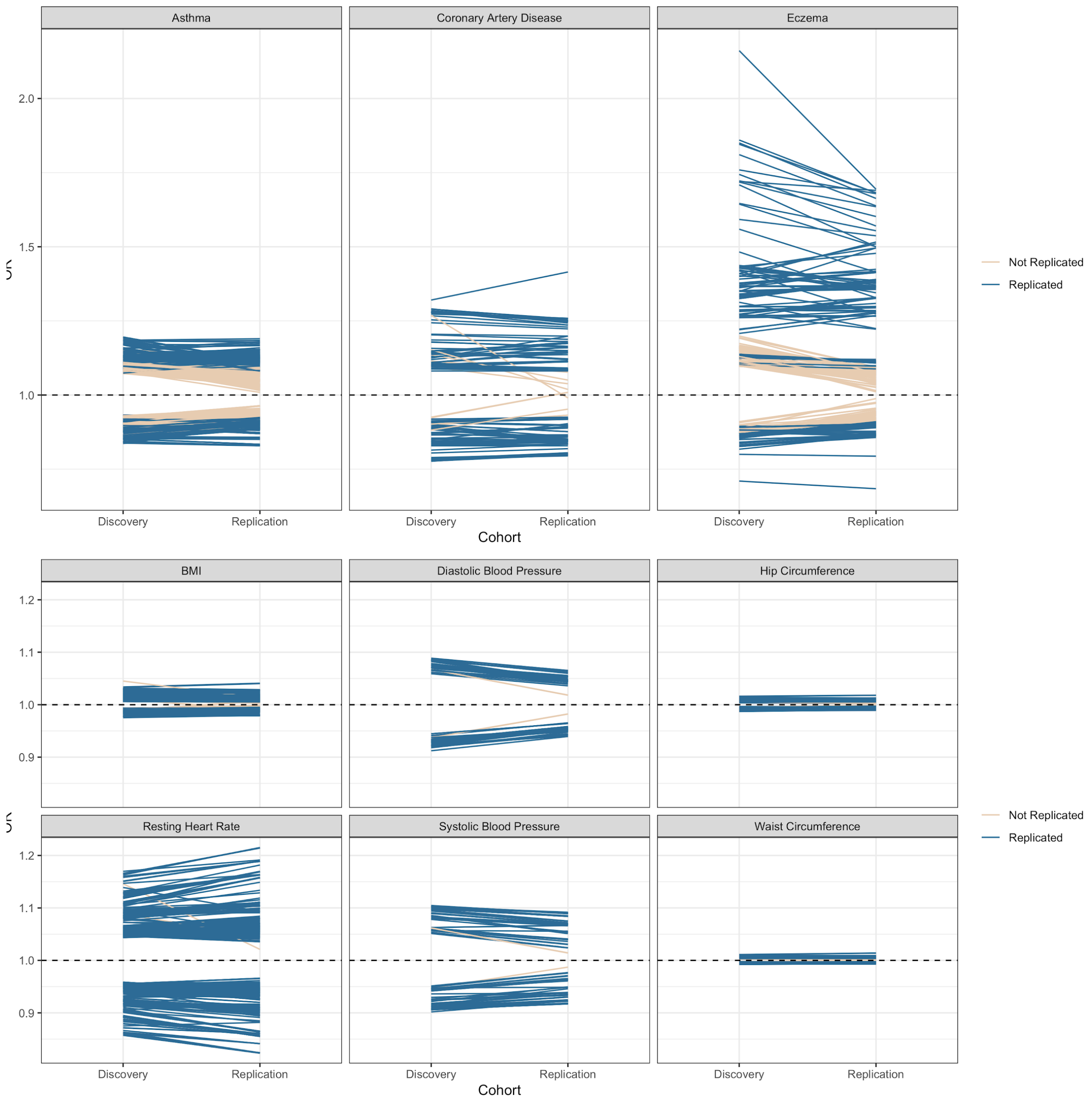

<u>eFigure 7</u>: SNV replication across phenotypes per Odds Ratio

Supplementary Tables

eTable1

eTable1: Sample size and replication rate across phenotypes

Disease	Total sample size	Discovery GWAS Cases	Discovery GWAS Controls	Replication GWAS Cases	Replication GWAS Controls	Number of Genome-wide significant SNVs	Number of SNVs that are replicated (%)
Asthma	225309	19954	107715	12362	85278	889	494 (56%)
SBP	430797	69395	N/A	361402	N/A	110	107 (97%)
Eczema	330142	10788	30047	9831	279476	640	337 (53%)
BMI	613900	234069	N/A	379831	N/A	1835	1756 (96%)
Waist Circumference	618033	232101	N/A	385932	N/A	937	827 (89%)
Hip circumference	598925	213038	N/A	385887	N/A	1083	1043 (96%)
Coronary Artery Disease/IHD	387786	22233	64762	14456	286335	159	149 (94%)
Resting Heart rate/Pulse Rate	447198	85787	N/A	361411	N/A	549	547 (99%)
DBP	430806	69395	N/A	361411	N/A	87	83 (95%)

eTable2

eTable2: Predictors of SNV replication

Predictor	P-value
Discovery GWAS SNV OR	<0.01
Discovery GWAS SNV P-value (continuous)	0.3
Discovery GWAS SNV P-value <5e-9 & >5e-10	0.6
Discovery GWAS SNV P-value <5e-10 & >5e-11	<0.01
Discovery GWAS SNV P-value <5e-11	<0.01
Quantitative trait	<0.01
Minor Allele Frequency (MAF)	<0.01

Imputation quality (INFO score)	<0.01
Sample size ratio	<0.01

eTable3

eTable3: Replication rate using more lenient, Bonferroni-corrected P-value thresholds

Phenotype	New P-value using P= 0.05/N	Replication % with P=0.05/n	Original replication %
Asthma	5.62E-05	77.9%	55.6%
SBP	4.55E-04	97.3%	97.3%
Eczema	7.81E-05	80.0%	53.0%
BMI	2.72E-05	98.0%	95.7
Waist Circumference	5.34E-05	98.7%	88.3%
Hip circumference	4.62E-05	98.9%	96.3%
Coronary Artery Disease/IHD	3.14E-04	95.6%	93.7%
Resting Heart rate/Pulse Rate	9.11E-05	99.8%	99.6%
DBP	5.75E-04	100%	95%

Bonferroni-corrected P-value thresholds calculated by P= 0.05/N, where N is the number of significant SNVs in discovery GWAS, used of using 5e-8)

eTable4

eTable4: Replication rate using P<10e-6

Disease	Total sample size	Number of Genome-wide significant SNVs using P<10e-6	Number of SNVs that are replicated* (%)
Asthma	225,309	2018	956 (47%)
SBP	430,797	536	489 (91%)
Eczema	330,142	1731	554 (32%)
BMI	613,900	4378	3834 (88%)
Waist Circumference	618,033	2598	2041 (79%)
Hip circumference	598,925	2882	2529 (88%)
Coronary Artery Disease/IHD	387,786	500	290 (58%)
Resting Heart rate/Pulse Rate	447,198	1301	1196 (92%)
DBP	430,806	877	641 (73%)

*Using P<10e-6

PRISM Checklist

Section and Topic	Item #	Checklist item	Location where item is reported
TITLE			
Title	1	Identify the report as a systematic review.	NR
ABSTRACT			
Abstract	2	See the PRISMA 2020 for Abstracts checklist.	Page 2
INTRODUCTION			
Rationale	3	Describe the rationale for the review in the context of existing knowledge.	Page 4
Objectives	4	Provide an explicit statement of the objective(s) or question(s) the review addresses.	Page 4

METHODS			
Eligibility criteria	5	Specify the inclusion and exclusion criteria for the review and how studies were grouped for the syntheses.	Page 5,6
Information sources	6	Specify all databases, registers, websites, organisations, reference lists and other sources searched or consulted to identify studies. Specify the date when each source was last searched or consulted.	Page 5,6
Search strategy	7	Present the full search strategies for all databases, registers and websites, including any filters and limits used.	Page 5, 6
Selection process	8	Specify the methods used to decide whether a study met the inclusion criteria of the review, including how many reviewers screened each record and each report retrieved, whether they worked independently, and if applicable, details of automation tools used in the process.	Page 5,6
Data collection process	9	Specify the methods used to collect data from reports, including how many reviewers collected data from each report, whether they worked independently, any processes for obtaining or confirming data from study investigators, and if applicable, details of automation tools used in the process.	Page 5,6
Data items	10a	List and define all outcomes for which data were sought. Specify whether all results that were compatible with each outcome domain in each study were sought (e.g. for all measures, time points, analyses), and if not, the methods used to decide which results to collect.	Page 5,6
	10b	List and define all other variables for which data were sought (e.g. participant and intervention characteristics, funding sources). Describe any assumptions made about any missing or unclear information.	Page 5,6
Study risk of bias assessment	11	Specify the methods used to assess risk of bias in the included studies, including details of the tool(s) used, how many reviewers assessed each study and whether they worked independently, and if applicable, details of automation tools used in the process.	NR
Effect measures	12	Specify for each outcome the effect measure(s) (e.g. risk ratio, mean difference) used in the synthesis or presentation of results.	Page 5,6,7,8
Synthesis methods	13a	Describe the processes used to decide which studies were eligible for each synthesis (e.g. tabulating the study intervention characteristics and comparing against the planned groups for each synthesis (item #5)).	Page 5,6,7,8
	13b	Describe any methods required to prepare the data for presentation or synthesis, such as handling of missing summary statistics, or data conversions.	Page 5,6,7,8
	13c	Describe any methods used to tabulate or visually display results of individual studies and syntheses.	Page 5,6,7,8
	13d	Describe any methods used to synthesize results and provide a rationale for the choice(s). If meta-analysis was performed, describe the model(s), method(s) to identify the presence and extent of statistical heterogeneity, and software package(s) used.	Page 5,6,7,8
	13e	Describe any methods used to explore possible causes of heterogeneity among study results (e.g. subgroup analysis, meta-regression).	Page 5,6,7,8
	13f	Describe any sensitivity analyses conducted to assess robustness of the synthesized results.	Page 5,6,7,8
Reporting bias assessment	14	Describe any methods used to assess risk of bias due to missing results in a synthesis (arising from reporting biases).	NR

Certainty assessment	15	Describe any methods used to assess certainty (or confidence) in the body of evidence for an outcome.	NR
RESULTS			
Study selection	16a	Describe the results of the search and selection process, from the number of records identified in the search to the number of studies included in the review, ideally using a flow diagram.	Page 9,10,11
	16b	Cite studies that might appear to meet the inclusion criteria, but which were excluded, and explain why they were excluded.	Page 9,10,11
Study characteristics	17	Cite each included study and present its characteristics.	Page 9,10,11
Risk of bias in studies	18	Present assessments of risk of bias for each included study.	NR
Results of individual studies	19	For all outcomes, present, for each study: (a) summary statistics for each group (where appropriate) and (b) an effect estimate and its precision (e.g. confidence/credible interval), ideally using structured tables or plots.	Page 9,10,11
Results of syntheses	20a	For each synthesis, briefly summarise the characteristics and risk of bias among contributing studies.	NR
	20b	Present results of all statistical syntheses conducted. If meta-analysis was done, present for each the summary estimate and its precision (e.g. confidence/credible interval) and measures of statistical heterogeneity. If comparing groups, describe the direction of the effect.	Page 9,10,11, Appendix
	20c	Present results of all investigations of possible causes of heterogeneity among study results.	Page 9,10,11, Appendix
	20d	Present results of all sensitivity analyses conducted to assess the robustness of the synthesized results.	Page 9,10,11, Appendix
Reporting biases	21	Present assessments of risk of bias due to missing results (arising from reporting biases) for each synthesis assessed.	NR
Certainty of evidence	22	Present assessments of certainty (or confidence) in the body of evidence for each outcome assessed.	NR
DISCUSSION			
Discussion	23a	Provide a general interpretation of the results in the context of other evidence.	Page 11,12,13,14
	23b	Discuss any limitations of the evidence included in the review.	Page 11,12,13,14

	23c	Discuss any limitations of the review processes used.	Page 11,12,13,14
	23d	Discuss implications of the results for practice, policy, and future research.	Page 11,12,13,14
OTHER INFORMATION			
Registration and protocol	24a	Provide registration information for the review, including register name and registration number, or state that the review was not registered.	NR
	24b	Indicate where the review protocol can be accessed, or state that a protocol was not prepared.	NR
	24c	Describe and explain any amendments to information provided at registration or in the protocol.	NA
Support	25	Describe sources of financial or non-financial support for the review, and the role of the funders or sponsors in the review.	Page 14,15
Competing interests	26	Declare any competing interests of review authors.	Page 14, 15
Availability of data, code and other materials	27	Report which of the following are publicly available and where they can be found: template data collection forms; data extracted from included studies; data used for all analyses; analytic code; any other materials used in the review.	Page 6

References

1. Watanabe K, Stringer S, Frei O, et al. A global overview of pleiotropy and genetic architecture in complex traits. *Nat. Genet.* 2019;51(9):1339–1348.

2. Zhu Z, Zhang F, Hu H, et al. Integration of summary data from GWAS and eQTL studies predicts complex trait gene targets. *Nat. Genet.* 2016;48(5):481–487.

3. Chinn S. A simple method for converting an odds ratio to effect size for use in meta-analysis. *Stat. Med.* 2000;19(22):3127–3131.
